# Supplementary material for: Neocortical pyramidal neurons with axons emerging from dendrites are frequent in non-primates, but rare in monkey and human
Source: eLife. 2022 Apr 20;11:e76101. doi: 10.7554/eLife.76101 (PMC9159751; doi:10.7554/eLife.76101)
Supplement: Transparent reporting form [file elife-76101-transrepform1.docx]

***eLife’s* transparent reporting form**

We encourage authors to provide detailed information *within their submission* to facilitate the interpretation and replication of experiments. Authors can upload supporting documentation to indicate the use of appropriate reporting guidelines for health-related research (see [EQUATOR Network](http://www.equator-network.org/%20)), life science research (see the [BioSharing Information Resource](https://biosharing.org/)), or the [ARRIVE guidelines](http://www.plosbiology.org/article/info:doi/10.1371/journal.pbio.1000412) for reporting work involving animal research. Where applicable, authors should refer to any relevant reporting standards documents in this form.

If you have any questions, please consult our Journal Policies and/or contact us: [editorial@elifesciences.org](mailto:editorial@elifesciences.org).

**Sample-size estimation**

- You should state whether an appropriate sample size was computed when the study was being designed
- You should state the statistical method of sample size computation and any required assumptions
- If no explicit power analysis was used, you should describe how you decided what sample (replicate) size (number) to use

Please outline where this information can be found within the submission (e.g., sections or figure legends), or explain why this information doesn’t apply to your submission:

We believe this information does not apply to our submission. We obtained from most of the the individual cases four values: the percentage of neurons having an AcD from total neurons assessed and second, percentages in supra- and in infragranular layers, and as requested the percentage of shared root cells.

Monkey and human material, or biocytin material has been limited. Basically we analysed what has been available to us. We now include 5 more cats with biocytin labeling. The “replicates” have been re-assessments using different methods (Golgi-Cox, Golgi-Kopsch, immunoflurescence, biocytin, Lucifer Yellow, genetic labeling). No extra animals had been sacrificed for our study. For a majority of cases/stainings, we assessed quite substantial numbers of neurons. Overall, we assessed >35.000 pyramidal cells in 56 individuals of 7 species.

We attempted to have minimum 5 individuals per group (taxon or order) to be able to run non-parametric tests.

**Replicates**

- You should report how often each experiment was performed
- You should include a definition of biological versus technical replication
- The data obtained should be provided and sufficient information should be provided to indicate the number of independent biological and/or technical replicates
- If you encountered any outliers, you should describe how these were handled
- Criteria for exclusion/inclusion of data should be clearly stated
- High-throughput sequence data should be uploaded before submission, with a private link for reviewers provided (these are available from both GEO and ArrayExpress)

Please outline where this information can be found within the submission (e.g., sections or figure legends), or explain why this information doesn’t apply to your submission:

We believe this information does not apply in full to our submission. Every individual case has been analysed once. We used different labeling methods which basically confirmed the proportions, e.g. biocytin tracing with counting at a light microscope, and immunofluorescent double labeling followed by confocal assessment of the Z-stacks.

We aimed at minimum 5 individuals (= biological replicates) per group (species or order) in order to be able to do a non-parametric test.

All numbers obtained are reported in the Tables. These numbers are in the source data files.

No technical replicates have been done. No outliers have been taken out.

Criteria for inclusion:

*“We could do only do 1 pig and 1 cat for the laminar analysis because the immunofluorescence did not to deliver sufficient basal dendritic SMI-32 labeling of supragranular neurons in the second available individual. Thus, for these two cases no reliable laminar data could be obtained.”*This is stated in the chapter “Immunofluorescence”.

Similarly, our mouse data are only from layer V because the genetic labeling has been restricted to large pyramidal neurons.

*“We assessed all pyramidal cells with sufficiently well stained basal and apical dendrites that had a recognizable axon. We analyzed fields of view where the labeled pyramidal cells are fairly perpendicularly oriented such that the apical dendritic trunk and the descending axon could be clearly seen.”* This has been stated in the chapter “Assignment of AcD”.

**Statistical reporting**

- Statistical analysis methods should be described and justified
- Raw data should be presented in figures whenever informative to do so (typically when N per group is less than 10)
- For each experiment, you should identify the statistical tests used, exact values of N, definitions of center, methods of multiple test correction, and dispersion and precision measures (e.g., mean, median, SD, SEM, confidence intervals; and, for the major substantive results, a measure of effect size (e.g., Pearson's r, Cohen's d)
- Report exact p-values wherever possible alongside the summary statistics and 95% confidence intervals. These should be reported for all key questions and not only when the p-value is less than 0.05.

Please outline where this information can be found within the submission (e.g., sections or figure legends), or explain why this information doesn’t apply to your submission:

We aimed at minimum 5 individuals (= biological replicates) per group in order to be able to do a non-parametric test. As can be seen in Figure 3B, we had 16 individuals in the “all primate” group versus 13 individuals in the “all non-primate” group. We had in total 13 human and 8 macaque individuals.

All requested information is in Source data files 1 and 2.

The type of statistical test and the p values are given and in the legend to Fig. 3B, in new Fig. 4, new Fig. 5, and Fig. 6.

(For large datasets, or papers with a very large number of statistical tests, you may upload a single table file with tests, Ns, etc., with reference to sections in the manuscript.)

**Group allocation**

- Indicate how samples were allocated into experimental groups (in the case of clinical studies, please specify allocation to treatment method); if randomization was used, please also state if restricted randomization was applied
- Indicate if masking was used during group allocation, data collection and/or data analysis

Please outline where this information can be found within the submission (e.g., sections or figure legends), or explain why this information doesn’t apply to your submission:

not applicable; this information does not apply to our submission.

**Additional data files (“source data”)**

- We encourage you to upload relevant additional data files, such as numerical data that are represented as a graph in a figure, or as a summary table
- Where provided, these should be in the most useful format, and they can be uploaded as “Source data” files linked to a main figure or table
- Include model definition files including the full list of parameters used
- Include code used for data analysis (e.g., R, MatLab)
- Avoid stating that data files are “available upon request”

Please indicate the figures or tables for which source data files have been provided:

We uploaded (xls files) Source data 1 for Figures 3A, 3B;

Source data 2 for Figure 4A,B;

Source data 3 for Figure 5,

Spurce data 4 for Figure 6

Source data 5 for Figure 7.

Data of all individuals are also reported in the supplementary Tables 1, 2, 3 in order to document the interindividual variability.

Model definition files: n.a.

Code: n.a.
